# Letter to the Editor: reply to “Comparison of patient setup accuracy for optical surface-guided and X-ray-guided imaging with respect to the impact on intracranial stereotactic radiotherapy”

**DOI:** 10.1007/s00066-024-02223-9

**Published:** 2024-04-05

**Authors:** Adi Robinson

**Affiliations:** Department of Radiation Oncology, AdventHealth Celebration, 380 Celebration Place Suite 100, 34747 Celebration, FL USA

Dear Editor,

I have read with great interest the original article entitled “Comparison of patient setup accuracy for optical surface-guided and X-ray-guided imaging with respect to the impact on intracranial stereotactic radiotherapy” by Schöpe, M., Sahlmann, J., Jaschik, S. et al. published in *Strahlentherapie und Onkologie* (November 2023). The paper examines the patient position accuracy of a surface-guided radiation therapy (SGRT) system (C-Rad) and compares it with an X‑ray-based imaging system (IGRT; BrainLab Exactrac) with an emphasis on stereotactic radiosurgery.

The article states that SGRT has recently been used in cranial stereotactic radiotherapy (SRT) but publications on the matter are sparse and primarily involve phantom studies. SGRT has been used in cranial radiosurgery for over 10 years with multiple publications using real patient data for the AlignRT system (Vision RT, London, UK) [1–3]. Furthermore, the paper states that currently, it is insufficiently known whether the accuracy of SGRT during non-coplanar treatments meets the requirements for SRT treatments. Again, multiple publications have shown SGRT (AlignRT) to have submillimeter accuracy even with couch rotations, head orientation, and multiple targets [1–15].

The authors make a few mentions of the specific SGRT system that was utilized in this study (C-Rad) but also use the generic term “SGRT” or “SGRT system,” which has broader implications. Currently there are at least four commercially available SGRT systems: AlignRT (Vision RT, London, UK), Catalyst HD (C-Rad, Uppsala, Sweden), IDENTIFY (Varian, Palo Alto, CA, USA), and Exactrac Dynamic (Brainlab AG, Munich, Germany) [16]. Each SGRT system has different specifications and performance and care should therefore be taken when making conclusions on one generic term. The abstract and conclusions of this article use the term “SGRT system” to describe the study and its results, which, based on many other publications, is misleading to the readers and I therefore recommend should be corrected.

For example, the article recommends increasing the treatment margin: “Compared to the ExacTrac® IGRT system, the SGRT system exhibits greater uncertainty in patient positioning during cranial irradiation, especially with non-coplanar fields. This is in the order of about 5 mm (P95). This means that when only using an SGRT system for positioning, a safety margin of 5 mm or 6 mm when creating the PTV is necessary to safely cover the clinical target volume (CTV). Otherwise, it cannot be assured that the CTV will receive the intended dose.” This would be significantly above the margins used for SRS treatments and exceed the ESTRO-ACROP/AAPM-TG302 guidelines for SRS/SBRT procedures, [16, 17] and is not consistent with the experience of other SGRT systems, as noted above.

Finally, the SGRT references used in the article are within the scope of the study performed; however, they refer to a different SGRT system (AlignRT) than used in the study (C-Rad). Previous studies have compared the C‑Rad SGRT system with respect to SRT/SRS and I believe those would be more relevant [17–20].
